# Monolithic 3D Oscillatory Ising Machine Using Reconfigurable FeFET Routing for Large‐Scalability and Low‐Power Consumption

**DOI:** 10.1002/advs.202413247

**Published:** 2025-03-16

**Authors:** Joon Pyo Kim, Song‐Hyeon Kuk, Hyun Wook Kim, Jaeyong Jeong, Juhyuk Park, Bong Ho Kim, Jongmin Kim, Aida Todri‐Sanial, Sanghyeon Kim

**Affiliations:** ^1^ School of Electrical Engineering Korea Advanced Institute of Science and Technology (KAIST) 291 Daehak‐ro Yuseong‐gu Daejeon 34141 Republic of Korea; ^2^ School of Electronic and Electrical Engineering Eindhoven Technical University Eindhoven AZ 5612 The Netherlands; ^3^ Device Technology Division Korea Advanced Nano Fab Center (KANC) 109 Gwanggyo‐ro Yeongtong‐gu Suwon 16229 Republic of Korea

**Keywords:** fefet, ising machine, m3d, maxcut, oscillator

## Abstract

Ising machines are attractive for efficiently solving NP‐hard combinatorial optimization problems (COPs). In this work, a scalable monolithic‐3D (M3D) oscillatory Ising machine (OIM) is proposed using ferroelectric field‐effect transistors (FeFETs) serving as an in‐memory routing switch (RS) and bi‐stable resistor (biristor)‐based oscillators for the first time. The M3D OIM achieves low static power consumption while offering high reconfigurability. Through careful control of FeFET routing switches, weights of the Ising model are embedded in coupled biristors. The performance is validated through simulations and experiments, in successfully solving King's graph sub‐problems and the MaxCUT problem. By leveraging the intrinsic OIM features of parallel computing together with M3D integration, it is reported that the M3D OIM outperforms reported OIMs in scalability and speed. Such an approach provides new insights and significant potential for solving COPs.

## Graphical abstract

This paper introduces a novel M3D Oscillatory Ising Machine (OIM) utilizing FeFETs as in‐memory routing switches and biristors as oscillators. The system achieves precise control, scalability, and low power consumption, solving complex optimization problems like MaxCUT problem. By leveraging parallel computing, reconfigurability, and M3D architecture, this OIM offers advantages over existing approaches, promising broader applicability for combinatorial optimization problems across industries.

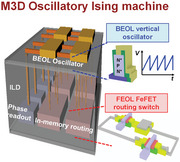

## 1 Introduction

Efficient combinatorial optimization problem (COP) solvers are of great interest due to their wide applications in various domains. Heuristic algorithms based on the conventional von Neumann architecture face limitations, particularly with the exponential increase in both power consumption and computing time as problem complexity grows.^[^ ^1^, ^2^, ^3^ ^]^ To address these limitations, physical Ising machines have been developed. These machines are designed to intrinsically accelerate the solution of many intractable COPs by directly minimizing the Ising Hamiltonian function *H*.^[^ ^4^, ^5^ ^]^ Existing Ising machines employ diverse physical mechanisms to solve COPs, each offering distinct advantages and trade‐offs. For instance, quantum annealers based on superconducting qubits inherently provide stochasticity through quantum tunneling effects, which can help escape local minima and explore the solution space effectively. However, the scalability of quantum annealers remains constrained by the need for cryogenic environments and the challenge of mitigating noise in large qubit systems.^[^ ^6^, ^7^, ^8^ ^]^ Optical Ising machines leverage the interference of light in degenerate optical parametric oscillators to emulate stochastic dynamics and parallelism. These machines achieve excellent speed and inherent randomness but face limitations in scalability due to the physical size of the long optical fibers and the complexity of maintaining stable optical coupling in large networks.^[^ ^9^, ^10^, ^11^ ^]^ In contrast, CMOS‐based digital annealers based on digital computation can offer excellent CMOS compatibility and enable innovative chip designs.^[^ ^12^, ^13^, ^14^ ^]^ However, these digital implementations tend to behave deterministically, making it difficult to achieve good solutions without additional stochastic random number generator circuits. While these technologies offer some advantages, their limitations in scalability and power consumption motivate the exploration of alternative approaches, such as oscillatory Ising machines (OIMs).

Oscillatory Ising machines are particularly attractive due to their inherent capability for parallel computing and their relatively simple design.^[^ ^15^, ^16^ ^]^ OIMs represent the nodes of the Ising model with the phase of coupled oscillators, forming a continuous dynamical system. As the system scales up, however, the oscillator phases can become scattered, making it difficult to distinguish between spin states. To address this issue, sub‐harmonic injection locking (SHIL) can be employed to force the oscillator phases into two distinct states, effectively stabilizing the system and enhancing its ability to identify the ground state of the Ising Hamiltonian.^[^ ^15^ ^]^ Nevertheless, as the complexity of the Ising model increases, the interconnections between nodes become more intricate, leading to scalability issues when embedding the model onto hardware. Specifically, as the number of nodes (*N*) in the Ising model grows, the number of coupling between them increases quadratically (≈*N* ^2^).^[^ ^17^ ^]^ Previously proposed large‐scale OIMs, especially those using CMOS ring oscillators and coupling routing switch (RS) as static random‐access memory (SRAM) with transmission gate cells, face challenges in achieving power‐efficient hardware due to their high static power during computing.^[^ ^18^ ^]^ Additionally, the scalability issue becomes severe as the proportion of the coupling elements and routing in the Ising machine rapidly increases, with the overhead in both area and power consumption far exceeding that of the oscillators themselves. Moreover, the parasitic capacitances caused by the increasingly complex routing wires lead to a significant drop in the oscillator frequency.^[^ ^19^ ^]^

**Figure 1:**
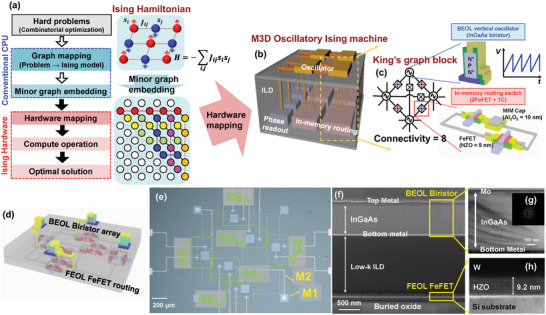
a) Workflow for solving combinatorial optimization problems using the Ising machine hardware. Complex combinatorial optimization problems are reformulated into an Ising Hamiltonian, which is then transformed into a format suitable for embedding onto the hardware. Once embedded in the Ising machine, the problem is iteratively solved to provide an optimized solution. b) The 3D schematic structure of the proposed monolithic 3D (M3D) integrated oscillatory Ising machine (OIM). c) The blocks of the M3D OIM are arranged in a King's graph configuration (connectivity = 8), where the edges consist of a two FeFETs‐one capacitor routing switch, and the vertices are represented by vertically stacked BEOL oscillators. d) 3D schematic illustration of the fabricated monolithic 3D integrated oscillatory Ising machine. The FEOL layer contains six FeFETs, and the BEOL layer includes four biristors stacked via wafer bonding, interconnected with the FeFETs. e) Optical microscope (OM) image of the fabricated FEOL FeFET routing. f) Cross‐sectional SEM image showing the stacking of the fabricated FEOL FeFET and BEOL biristors. TEM image of the g) BEOL InGaAs biristor gate stack and h) FEOL FeFET. The inset in (g) shows an electron diffraction image of the InGaAs layer.

In this work, we propose a highly scalable monolithic 3D (M3D) OIM, featuring the stacked oscillators in the BEOL on top of the front end of the line (FEOL) coupling elements, simplifying the routing structure and reducing parasitics caused by conventional complex routing. For routing functionality, Si ferroelectric field‐effect transistors (FeFETs) are used as coupling elements to enable fast reconfigurability and low static power consumption in the RS, controlling the coupling between oscillators (**Figure** **1**b). To solve COPs, the Ising model, which is a reformulated representation of COPs, is modified into a structure such as a King's graph via minor‐embedding heuristics.^[^ ^20^, ^21^ ^]^ This transformation is essential because directly embedding all connections of complex COPs onto hardware is practically infeasible. Additionally, as the number of coupling elements connected to a single node increases, parasitic effects also increase, further complicating hardware implementation. The embedded models are then mapped onto the Ising machine to solve the optimization problems (Figure 1a). For diverse problems to be solved on a single hardware platform, reconfigurability is essential to adjust the oscillator couplings dynamically. Therefore, we developed a hardware platform based on a King's graph with a connectivity of 8. This platform consists of four oscillators connected through six memory RS devices, which store the connectivity between the oscillators (Figure 1c). For the memory RS device, among various memory devices, FeFETs are novel devices that offer low‐leakage and fast programming capabilities, which provide the desired functionality for RS.^[^ ^22^, ^23^ ^]^ Moreover, compared to SRAM, FeFETs can provide significant advantages in terms of area efficiency and reduced static power consumption.^[^ ^24^ ^]^ To achieve a programmable coupling element, a series combination of a FeFET and a metal‐insulator‐metal (MIM) capacitor is used for capacitive coupling, and a FeFET is used for resistive coupling. These two couplings are connected in parallel, allowing coupling control based on the controllable resistance state of each FeFET. Furthermore, we used an oscillator with a back end of line (BEOL) compatible InGaAs bi‐stable resistor (biristor), which is a single crystalline semiconductor with an NPN junction device.^[^ ^25^ ^]^ The highly steep junction abruptness between the highly doped n^+^ and p layers induces impact ionization, which allows the use of a simple vertical structure as a voltage oscillator.^[^ ^26^ ^]^ First, we evaluated the electrical characteristics of the FEOL Si‐FeFET as an RS. Next, we assessed the electrical characteristics of the BEOL stacked biristor as an oscillator. Then, we systematically investigate the controllability of FeFET RS between the biristors. Various King's graphs minor‐graphs are embedded into four‐nodes six‐edges M3D OIM and their success probability is evaluated. Finally, we assess and report on the success probability and time‐to‐solution of M3D OIM in a large‐scale problem through the simulation of the MaxCUT problem using an annealing scheme.

## 2 Results and Discussion

### 2.1 M3D Integration of BEOL Oscillator on FEOL Si‐FeFET Routing Switch

Figure 1d shows a 3D schematic of the fabricated four‐node, six‐edge M3D OIM, and Figure 1e presents the optical microscope (OM) image of the fabricated FEOL FeFET routing. First, six FeFETs were fabricated on the FEOL, followed by the deposition of 10 nm Al_2_O_3_ deposited by atomic layer deposition (ALD), which was used as the dielectric for the MIM capacitor. Subsequently, W metal 1(M1) was deposited for the MIM capacitor and the connection of the five RS devices. The capacitance of the Al_2_O_3_ MIM capacitor was determined to be ≈1.1 × 10^−14^ F µm^−2^, as shown in the capacitance–voltage curve in Figure S1 (Supporting Information). To achieve various capacitance values, we adjusted the area based on the normalized capacitance values of the MIM capacitors to experimentally observe the coupling behavior of the oscillators at different capacitance values. To connect the oscillators in the form of a King's graph, the diagonal oscillators must be interconnected. In doing so, one RS must be electrically isolated from the rest of the diagonal switches. For this purpose, 100 nm of PE‐TEOS was deposited and M2 was deposited after via hole etching. Then, an interlayer dielectric (ILD) was deposited for the M3D integration. For M3D integration, metal wafer bonding technology was employed. After depositing bonding metal on the epitaxially grown InGaAs wafer, which consists of collector, base, and emitter layers for the BEOL biristor, the InGaAs wafer was directly bonded onto the fabricated FEOL FeFET wafer. Following the fabrication of the BEOL biristor, interconnection vias were etched, and the metal connections were formed to provide vertical interconnects. The detailed schematic of the M3D OIM fabrication process is shown in Figure S2 (Supporting Information) and the Experimental Section. Figure 1f shows the cross‐sectional scanning electron microscope (SEM) image of the fabricated M3D OIM, indicating the fully processed 2nd‐tier M3D integrated structure. Figure 1g,h show the transmission electron microscope (TEM) image of the BEOL biristor and gate stack of the FEOL Si‐FeFET, respectively, demonstrating the high crystal quality of the n^+^pn^+^ InGaAs layer and crystallized HZO.

**Figure 2:**
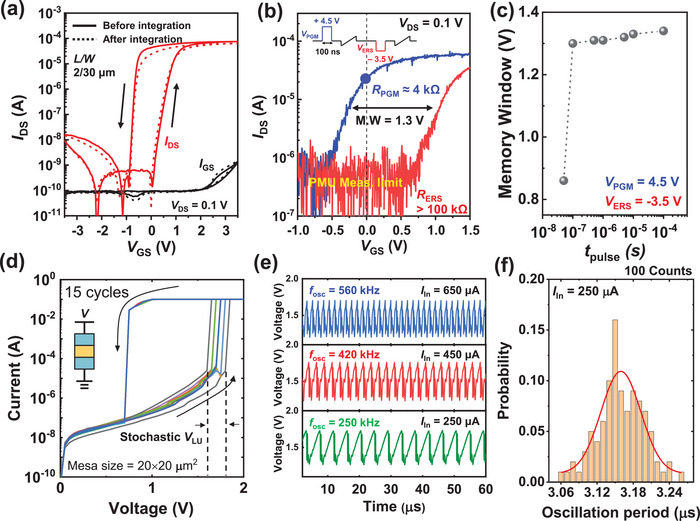
a) DC *I*_DS_‐*V*_GS_ curve with ±3.5 V dual sweep of the FEOL Si FeFET before and after integration of the BEOL biristor device. b) Pulsed *I*_DS_‐*V*_GS_ curve of the FeFET device using a single PGM/ERS pulse scheme in the inset. c) Memory window of the FeFET according to the write pulse time (*t*_pulse_). *t*_pulse_ > 100 ns shows memory window > 1 V. d) Hysteric DC *I–V* curve of the biristor with 15 cycles. Stochastic latch‐up voltage is shown during operation. e) Output voltage oscillation waveforms according to the different input currents (*I*_in_) to the biristor. f) Measured oscillation periods during *I*_in_ = 250 µA, showing jitter noise in the biristor.

First, to evaluate the performance of the RS, we examined the electrical characteristics of the FEOL Si‐FeFET in the M3D OIM. **Figure** **2**a shows the ±3.5 V DC dual sweep *I*_DS_‐*V*_GS_ characteristic with the *V*_DS_ = 0.1 V of the fabricated FEOL FeFET before and after integration of the top biristor, showing a low leakage current and no degradation in the memory window (MW) after the integration process. This result highlights that the low fabrication temperature (<100 °C) of the BEOL biristor caused no degradation to the FEOL FeFET device. Pulsed *I*_DS_‐*V*_GS_ measurements in Figure 2b were also conducted to characterize the switch operation of the FeFET device. When the FeFET was programmed with the program (PGM) and erase (ERS) pulse voltage of +4.5 V, and −3.5 V, respectively, and pulse time of 100 ns, *R*_PGM_ = 4 kΩ and the *R*_ERS_ > 100 kΩ were achieved. These distinct resistive states will be used to control the weight of the M3D OIM. Figure 2c shows the dependence of the MW on the program pulse width, revealing that a fast write speed of 100 ns was achieved while maintaining an MW of 1.3 V. This indicated that the fabricated Si FeFET can achieve a large MW with short switching pulses, making it an ideal RS device with fast computation time and low power consumption. Additionally, the PGM and ERS states show clear separation as measured across the 35 devices, confirming that they are adequate for use in large RS arrays (Figure S3, Supporting Information). Furthermore, memory retention exceeding 10^3^ s was observed in Figure S4 (Supporting Information), indicating that the RS can maintain its stored resistance long enough to complete oscillator‐based computation after PGM/ERS.

Next, we evaluated the electrical characteristics of the BEOL biristor‐based oscillator integrated on top of the FEOL FeFET. Figure 2d shows the measured hysteric *I*–*V* characteristic of the fabricated BEOL biristor with a mesa size of 20 × 20 µm^2^. As the voltage increases and reaches the threshold latch‐up voltage (*V*_LU_) voltage, the current latches up due to the impact ionization at the abrupt junction between the base and collector regions, and it latches down when the voltage decreases back below the threshold. Notably, *V*_LU_ exhibits stochastic behavior due to the random nature of the impact ionization,^[^ ^27^ ^]^ which favorably helps to escape from local minima during OIM computing. Figure 2e shows the measured voltage output waveforms of the biristor at various input currents (*I*_in_), showing that the speed of the oscillator is fully controlled by the *I*_in_. The principle of voltage oscillation due to impact ionization when the input current is biased has been described in Figure S5 (Supporting Information). Additionally, Figure 2f shows an oscillation period of 100 counts during *I*_in_ = 250 µA, highlighting how the stochasticity in *V*_LU_ results in variations in the oscillation period. Since the frequency of the biristor (*f*_osc_) increases proportionally with capacitor scaling, *f*_osc_ is expected to exceed GHz operation when the parasitic capacitor of the biristor is scaled down, as shown in Figure S6 (Supporting Information).

### 2.2 Reconfigurable Coupling Functions Between Oscillators Using FeFET Routing

**Figure 3:**
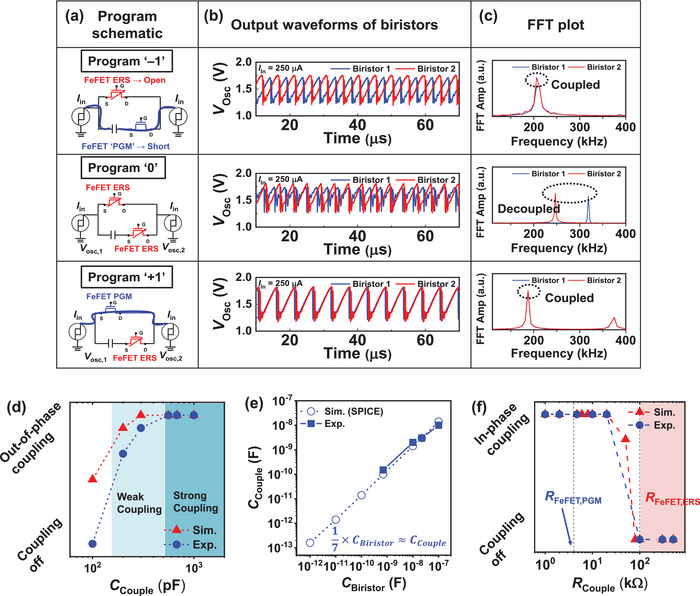
a) Program weight schematic of the two FeFET‐one capacitor routing switches between two biristors. b) Output waveforms and c) FFT plots of the two biristors according to the programmed FeFET routing. d) Probability of being out‐of‐phase coupling according to the coupling capacitor. e) Coupling capacitor trend according to the capacitance of the biristor. f) Probability of being in‐phase coupling according to the coupling resistor.

To embed various graphs in the hardware, the coupling weight of the oscillators should be adjustable. To verify the reconfigurability of the FeFET RS between the oscillators, RS was programmed as shown in **Figure** **3**a with weights (*w*_ij_) of −1, 0, and +1, respectively. When the FeFET connected with the capacitor is in the PGM state and the opposite FeFET is in the ERS state, capacitive coupling between the two oscillators occurs, showing out‐of‐phase coupling (*w*_ij_ = −1).^[^ ^28^, ^29^ ^]^ When both FeFETs are in the ERS state, the oscillators are decoupled and oscillate independently (*w*_ij_ = 0). When the FeFET connected with the capacitor connected is in the ERS state and the opposite FeFET is in the PGM state, the two oscillators show in‐phase coupling, acting as a single node (Figure 3b). The presence or absence of coupling between two biristors can also be verified via the quantified fast Fourier transform (FFT) plot in Figure 3c. When the biristors are coupled and exhibit a certain weight, their FFT peaks appear at the same frequency. However, when they are decoupled, the FFT peaks of the two biristors appear at different frequencies. The results demonstrate that when the two biristors are connected via capacitive or resistive coupling, their FFT peaks align at the same frequency. Conversely, when all FeFETs are in the ERS state and the connection is broken, the biristor‐based oscillators show independent FFT peaks. Notably, capacitive/resistive coupling slows down the oscillators compared to their original frequencies due to the loading effect caused by the coupling element.^[^ ^19^ ^]^ These measurements experimentally confirm that the fabricated M3D OIM can input three distinct weights via the RS, which is an important function for embedding various Ising models in the hardware platform. Additionally, the voltage difference across the coupling FeFET caused by the oscillators is a maximum of 0.6 V. At this voltage level, it was confirmed that neither the programmed resistance state of the FeFET nor its parasitic capacitance is affected, ensuring that the coupling remains unaffected.

To systematically investigate the probability of coupling configurations, we measured the coupled biristors while adjusting the coupling strength. When the coupling capacitor (*C*_C_) is set to 150 pF, the probability of out‐of‐phase is dominant, whereas, above 500 pF, only out‐of‐phase coupling is observed (Figure 3d). However, the strong coupling can make it difficult for the system to reach the global minimum, so we used the weakly coupled region in the following experiment.^[^ ^30^ ^]^ In addition, when the parasitic capacitor of the biristor is scaled, the value of *C*_C_ needed to induce out‐of‐phase coupling also scales, highlighting the scalability of our Ising solver architecture (Figure 3e). The impact of the coupling resistor (*R*_C_) was also investigated. The results in Figure 3f show that the two biristors exhibit in‐phase coupling up to *R*_C_ = 20 kΩ, and for the larger *R*_C_, the biristors are not coupled. Notably, the resistance of FeFET in PGM (*R*_PGM_) and ERS (*R*_ERS_) states corresponds to 4 kΩ and > 100 kΩ, respectively, indicating that the fabricated FeFET is an appropriate candidate to serve as a switch that can turn coupling on and off. Figure S7 (Supporting Information) shows the impact of the *R*_C_ value on the settling time/cycles until the solution when an injection locking signal is applied. The results indicate that there is no significant difference in settling cycles depending on the *R*_C_, suggesting that the value of *R*_PGM_ does not change the settling time of the Ising machine.

### 2.3 Solving MaxCUT Problem Using M3D Oscillatory Ising Machine Routed by FeFET

**Figure 4:**
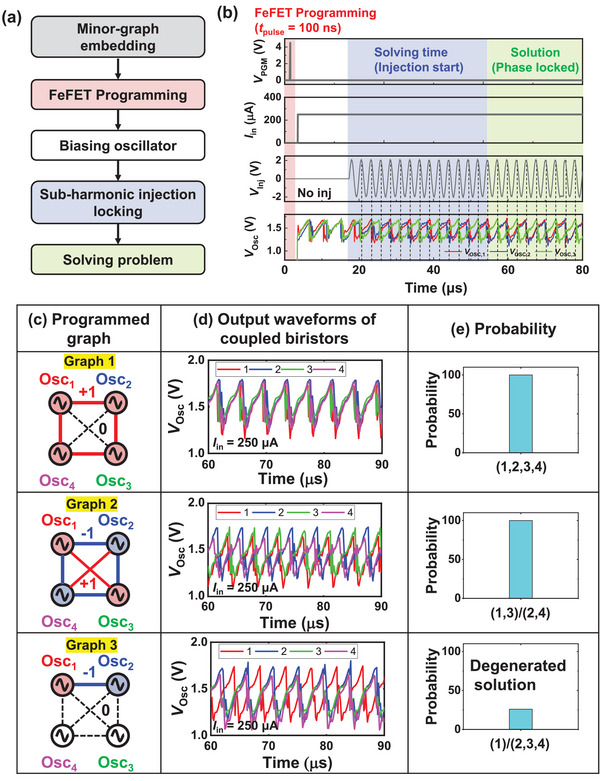
a) Process schematic and b) timing diagram for input and output electrical signals during solving Ising problems with the M3D oscillatory Ising machine. c) Programmed weight schematic of the six‐routing switches and four‐biristors King's graph block. d) The measured output waveforms of the biristors and e) probability of 50 runs according to the mapped graphs.

Based on the experimental results, we experimentally mapped sub‐graphs of the MaxCUT problem onto the M3D OIM, which is one of the famous NP‐hard problems that can be directly mapped onto the Ising model.^[^ ^31^ ^]^ The experimental measurement setup for the M3D OIM is shown in Figure S8 (Supporting Information), and the schematic for solving the problem is shown in **Figure** **4**a. First, the corresponding graph, divided into minor sub‐graphs by a heuristic, is embedded by programming the FeFETs in the RS. Then, the *I*_in_ is applied to the biristors to initiate voltage oscillation, producing a frequency of *f*_osc_. Immediately after, a sinusoidal SHIL signal with an injection voltage (*V*_inj_) = 2.0 V and an injection frequency (*f*_inj_) ≈2*f*_osc_ is applied to the biristors by the function generator (for more detail about the SHIL, refer to Figure S9 (Supporting Information). Finally, the resulting voltage oscillations in the biristors are captured by the oscilloscope. Figure 4b shows a timing diagram of the input and output signals during the operation of the M3D OIM. Approximately eight cycles after injection starts, the biristor waveforms lock into either in‐phase or out‐of‐phase states. As previously mentioned, the retention time of the weight embedded in the FeFETs is much longer than the computing time required for the oscillators to phase‐lock, ensuring stable operation of the M3D OIM. Using the six‐RS four‐biristor M3D OIM, we embedded various sub‐graphs, as shown in Figure 4c. In graph 1, the diagonal RSs are open, and the remaining four RSs are shortened to make the graph connected by the same node. The output waveforms of coupled biristors in Figure 4d show that the 4 biristors are coupled in‐phase, and the probability of running 50 runs was 100% (Figure 4e). In graph 2, the diagonal RSs were shorted to represent the same node, and the other four RSs were capacitively coupled to make the adjacent nodes have opposite phases. The output waveforms show that biristors 1 and 3, and biristors 2 and 4 are out‐of‐phase with each other with a probability of 100%. In graph 3, a graph was embedded in which only RS between adjacent nodes were capacitively coupled and all remaining RSs were open. The output waveforms of coupled biristors show that biristors 1 and 2 are out‐of‐phase and biristors 3 and 4 were randomly locked to the phase of biristors 1 or 2. The measured results indicate that the desired weights can be accurately reconstructed in the M3D OIM. Furthermore, as shown in Figure S10 (Supporting Information), additional graphs were tested using the M3D OIM, further validating the M3D OIM's reconfigurability and its ability to handle diverse graph configurations effectively.

**Figure 5:**
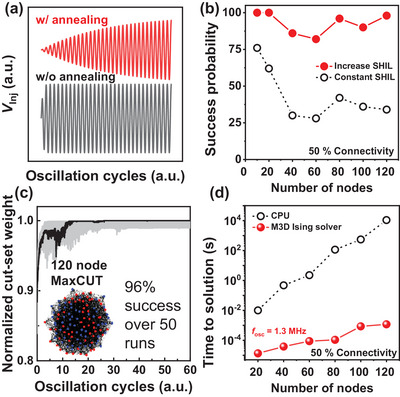
a) The annealing scheme is used during injection locking in the MaxCUT simulation. b) The success probability of 50 runs of the MaxCUT simulation, with and without the annealing scheme, as a function of the number of nodes in the MaxCUT problem. c) Simulation results of the normalized cut‐set weight for a 120‐node MaxCUT problem with 50% connectivity density over 50 runs. The light‐coloured curves show the result of 50 runs and the dark‐coloured curve shows one best simulation result. d) Comparison of the time to solution of the M3D oscillatory Ising machine and a CPU according to the number of nodes in the MaxCUT problem.

To further assess the feasibility and performance of the M3D OIM, we conducted a simulation based on stochastic differential equations solving the large‐scale MaxCUT problem.^[^ ^15^ ^]^ As the size of the graphs increases, the energy landscape becomes more complex, with numerous local minima forming, which increases the likelihood of getting stuck in these local minima rather than reaching the global minimum. Therefore, an annealing schedule is required to help escape from the local minima. The OIM can implement an annealing scheme by controlling the amplitude of the SHIL as we described in our previous work, and we investigated its impact on solution quality (**Figure** **5**a). The success probability of OIM is defined as the proportion of trials that successfully identified the true ground‐state energy relative to the total number of trials conducted. Here, the benchmark for the true ground state was established based on solutions obtained from the Biq Mac solver, which employs a branch‐and‐bound algorithm.^[^ ^32^ ^]^ The results in Figure 5b show that for a MaxCUT problem with 50% connectivity, using a constant SHIL results in a sharp drop in the success probability to below 50% as the number of nodes increases. This is due to the increased likelihood of getting stuck in local minima as the complexity of the coupled network grows with more nodes. In contrast, using an increasing SHIL annealing scheme improves the probability of escaping local minima, resulting in better solution quality in the M3D OIM. The cut‐set weight for 50 runs of 120 oscillators is depicted in Figure 5c, showing a 96% success probability over 50 runs. Moreover, the M3D OIM shows a much faster time to achieve the solution compared to the digital CPU, which clearly demonstrates the advantage of the M3D OIM (Figure 5d).

The M3D OIM exhibits superior performance compared to previously reported OIMs, as summarized in **Table** **1**. To the best of our knowledge, this work is the first demonstration of an M3D OIM, which is enabled by the use of biristors as oscillators—simple devices with a 4F^2^ footprint that are fabricated at process temperatures below 100 °C. Notably, when compared to conventional OIMs in terms of fabrication complexity, the M3D integration approach might need additional process steps such as wafer bonding, etc., but it can be done with a CMOS‐compatible process. Wafer bonding and 3D integration have been already typically used in CFET, and future CMOS architecture.^[^ ^33^ ^]^ As a result, it significantly reduces interconnect complexity and enhances scalability by simplifying the fabrication process. In contrast to traditional Ising chips, which must account for intricate planar routing, the vertical stacking achieved through M3D integration streamlines the fabrication process. The M3D structure also reduces parasitic load caused by wiring, which allows the oscillator frequency to be maintained more effectively compared to planar structures where the oscillator and coupling element exist in the same plane (Figure S11, Supporting Information). Additionally, while OIMs based on emerging devices such as ovonic threshold switching^[^ ^34^ ^]^ or insulator‐to‐metal transition (IMT) materials^[^ ^35^ ^]^ use passive elements as coupling components, limiting reconfigurability, this work presents a reconfigurable solution by employing FeFET‐based RS, ensuring both reconfigurability and scalability. Moreover, by utilizing in‐memory routing, the M3D OIM achieves lower standby power consumption compared to other CMOS‐based OIMs that rely on SRAM to store weights. Furthermore, due to the high‐speed operation of scaled biristors, the M3D OIM is expected to offer a fast time‐to‐solution and low power consumption even for complex MaxCUT problems.

**Table 1:** Benchmark of the M3D oscillatory Ising machine with other reported oscillatory Ising machines.

	CMOS ROSC ^[^ ^36^, ^37^ ^]^	Ovonic Threshold Switching Device ^[^ ^34^ ^]^	Phase Transition Device ^[^ ^19^ ^]^	MEMS Oscillator ^[^ ^38^ ^]^	This work
M3D Integration (BEOL compatibility)	× (×)	× (°)	×(△)	× (×)	°(°)
Implementation	>18 Transistors (9‐stage ROSC)	Chalcogenide switch (1T+1R)	VO_2_ device (1T+1R)	LiNbO_3_ resonator (1T+4R+MEMS)	Vertical biristor (1R)
Operating principle of the oscillator	CMOS inverter	Phase‐change switching	Insulator‐metal transition	MEMs Pierce oscillator	Single transistor latch
Cell area of single vertex and edge (F^2^)	>260	‐	‐	‐	∼60
Reconfigurability	°(SRAM)	×	×	×	°(FeFET)
Coupling	Resistive	Capacitive	Capacitive	Resistive	Capacitive/Resistive
Time to solution	23 µs	35 µs	30 µs	90 ns	1.11 µs
Energy to solution	924 nJ	2.3 µJ	76.8 nJ	–	5.5 nJ^*)^

^*)^ Calculated for projected oscillation frequency with size scaling.

## 3 Conclusion

We have proposed the novel M3D OIM based on an in‐memory RS with a FEOL FeFET device and BEOL biristors. Through careful evaluation of FeFET as RS and its impact on the coupled oscillators, we achieved precise control over three functions to embed the weights of the Ising model on the coupled biristors. The performance of the proposed M3D Ising machine was validated through experiments and simulations, successfully solving King's graph sub‐problems and MaxCUT. By taking advantage of parallel computing and the stochastic nature of OIM, we successfully implemented reconfigurability, a feature that other OIMs using emerging devices do not possess. Moreover, by using FeFETs as RSs and 4F^2^ biristors as oscillators, we achieved ultimate scalability and low power consumption in the device structural scaling using M3D for the first time. Building on this study, we plan to explore methods such as increasing the injection signal frequency to represent more diverse states and employing variable coupling elements to enable multi‐weight capabilities, thereby addressing more complex problems. We anticipate that our proposed solver will enhance its applicability, both at the device and structural levels, for solving increasingly complex COPs across a broad spectrum of industries and applications.

## 4 Experimental Section

### Epitaxial Growth

To obtain a high‐crystal quality InGaAs active layer, it was grown on an InP substrate using metalorganic vapor phase epitaxy (MOVPE) while maintaining a negligible lattice mismatch. To achieve a high doping concentration in the collector and emitter of the InGaAs, tellurium (Te)‐doped InGaAs was introduced using a diethyl telluride (DETe) precursor. To induce the latch effect in the InGaAs biristor, the emitter and collector were each grown to a thickness of 200 nm, while the base was grown to 400 nm. Furthermore, for the active layer transfer through wafer bonding, a 200 nm InGaAs and a 30 nm InP etch stop layer between the active layer and the substrate was inserted.

### Device Fabrication

Figure S2 (Supporting Information) shows the schematic of the detailed fabrication steps for the M3D OIM. First, a mesa of the silicon‐on‐insulator (SOI) wafer (channel thickness = 50 nm) was formed. Then, implantation (Arsenic, 30 keV) and activation annealing (1000 °C, 1 min) were carried out. Next, 9 nm Hf_0.5_Zr_0.5_O_2_ (HZO) was deposited by ALD after the removal of native oxide. Following this, gate metal formation was completed, and post‐metal annealing (PMA) was processed at 500 °C, 30 s. Then, source/drain metal formation was completed and PMA was also processed at 300 °C, for 10 min. Subsequently, 10 nm Al_2_O_3_ was deposited by ALD, and M1 was deposited for the MIM capacitor and connection of the RSs. Next, 100 nm of PE‐TEOS was deposited and M2 was deposited after via hole etching. Then, ILD was deposited for the M3D integration. For the fabrication of the BEOL biristor, the bonding metal of molybdenum (Mo)/gold (Au) with thicknesses of 20/25 nm was deposited on the InGaAs wafer and was directly bonded on the FEOL FeFET wafer. Subsequently, the InGaAs substrate was removed through wet etching with an H_3_PO_4_: H_2_O_2_:DI = 1:1:5 solution. To obtain the InGaAs channel, the InP etch stop layer was eliminated using a wet etch with an H_3_PO_4_:HCl = 3:1 solution. Next, a positive photoresist (PR) was patterned on the InGaAs layer, and again an H_3_PO_4_:H_2_O_2_:DI solution was used to etch the InGaAs layer, defining the mesa layer of the BEOL biristor. The underlying bonding metal and via contact with FEOL devices were also defined through PR patterning followed by wet etching and RIE dry etching. Subsequently, the native oxide in the InGaAs was removed using an NH_4_OH:DI solution, followed by sulfur passivation treatment in an (NH_4_)_2_S_x_: DI solution, which was known to effectively reduce defects and passivate the InGaAs. Finally, the top contact metal of the BEOL biristor and interconnection metal was deposited.

### Measurements

The SEM and TEM images were taken using Helios 5 UX (Thermofisher) and JEM‐ARM200F (JEOL), respectively. The DC *I‐V* electrical characteristics of the FeFET and InGaAs biristors were measured using a 4200A‐SCS parameter analyzer (Keithley). The pulse measurements were conducted by the ultra‐fast pulse measure unit 4225‐PMU (Keithley). Sinusoidal waveforms used for injection locking were generated by AFG 3022B dual channel arbitrary/function generator (Tektronix) and the waveforms were measured using a TBS 2204B oscilloscope (Tektronix).

### Simulation

SPICE simulations were conducted using HSpice (Synopsys) software. To model the InGaAs biristor, a voltage‐controlled switch was connected in parallel with a measured parasitic capacitor of 700 pF (the parasitic capacitor includes an inherent measurement setup). Furthermore, to demonstrate the hysteresis switching characteristics, parameters of *V*_th_ = 1.45 V, *V*_h_ = 0.25 V, *R*_on_ = 20 Ω, and *R*_off_ = 10^6^ Ω were utilized. To consider the wire parasitics in the simulation, line‐to‐line and line‐to‐ground metal parasitics were considered from the 28 nm PDK.

To address the MaxCUT problems through multiple simulation runs, a stochastic differential equation solver was employed within a MATLAB simulation framework to closely replicate the dynamics of the InGaAs biristor‐based oscillator system under injection locking. The waveform of the InGaAs biristor exhibits a sawtooth pattern with a long rise time and a short fall time as shown in Figure 2e. Therefore, to express this in an equation, the oscillation behavior of the biristor was modeled using the arcsin(tanh(.)) function. The fitting of both the coupling strength and the injection locking strength was aligned with the experimental observations. The magnitude of noise in the simulation model was based on the jitter noise of the InGaAs biristor‐based oscillator in Figure 2f.

## Conflict of Interest

The authors declare no conflict of interest.

## Author Contributions

J. P. Kim and S. H. Kim conceived the concept of this work. J. P. Kim and S ‐H. Kuk conducted the device fabrications. J. P. Kim conducted device characterizations and analysis. H. W. K conducted the circuit‐based simulation. J. Jeong, J. Park, B. H. Kim, and J. Kim contributed to the experiment and characterization methodology. S. H. Kim supervised the project. All authors wrote the manuscript.

## Supporting information



Supporting Information

## Data Availability

The data that support the findings of this study are available from the corresponding author upon reasonable request.
