# Supplementary material for: Monolithic 3D Oscillatory Ising Machine Using Reconfigurable FeFET Routing for Large‐Scalability and Low‐Power Consumption
Source: Adv Sci (Weinh). 2025 Mar 16;12(18):2413247. doi: 10.1002/advs.202413247 (PMC12079487; doi:10.1002/advs.202413247)
Supplement: Supplementary file 1 — Supporting Information [file ADVS-12-2413247-s001.docx]

Copyright WILEY-VCH Verlag GmbH & Co. KGaA, 69469 Weinheim, Germany, 2016.

Supplementary Information

**Title: Monolithic 3D Oscillatory Ising Machine using Reconfigurable FeFET Routing for large-scalability and low-power Consumption**

*Joon Pyo Kim, Song-Hyeon Kuk, Hyun Wook Kim, Jaeyong Jeong, Juhyuk Park, Bong Ho Kim, Jongmin Kim, Aida Todri-Sanial, and Sanghyeon Kim**

**Table S1. Benchmark of the oscillator-based Ising machine with other physical Ising solvers**.

|  | **Digital (Simulated annealing)^[1]^** | **D-wave 2000Q^[2]^** | **Coherent Ising machine^[3]^** | **Latch-based Ising machine^[4]^** | **Memristor-based Ising machine^[5]^** | **This work** |
| --- | --- | --- | --- | --- | --- | --- |
| **Architecture** | All-to-all | Sparse | All-to-all | Sparse | All-to-all | Sparse |
| **Ising spin** | Spins | Qubits | Coherent light | Latch voltage | Digital bits | Oscillator phases |
| **Implementation** | CPU | Compound Josephson-junction | Optical parametric oscillator | CMOS-latch | Memristor noise | Vertical biristor |
| **Scalability** | × | × (Cryogenic environment) | ×(long fiber ~ km) | O | O | O |
| **Time to solution** | 246 ms | > 10^4^ s (N = 55) | 2.3 ms | 45.8 μs (N = 2304) | 25 μs | 1.11 μs |
| **Energy to solution** | 14.8 J | > 250 MJ | > 460 mJ | 1.6 μJ | 400 nJ | 5.5 nJ^*^ |

^*^Calculated for projected oscillation frequency with size scaling

**Figure S1. Measured capacitance-voltage curve of the fabricated MIM capacitor with Al_2_O_3_ 10 nm.**

**Figure S2. Schematic of the fabrication process of the M3D oscillatory Ising machine.**

**Figure S3. The measured threshold voltage (*V*_th_) of the FeFET after program/erase operations across 35 devices. The clear separation of *V*_th_ demonstrates the device’s suitability for large-scale arrays.**

**Figure S4. Retention characteristics of the FeFET show no change in resistance for PGM, ERS states more than 10^3^ s.**

**Figure S5. The operation mechanism of the voltage oscillation property in the InGaAs biristor when a forbidden constant current is applied to the collector.**

Figure S5 shows the energy band diagram of the n^+^pn^+^ InGaAs junction to illustrate how oscillation characteristics work in the biristor. When the forbidden constant current (*I*_in_, the current level between the *V*_LU_ and *V*_LD_) is applied to the n^+^ collector, charges are time-integrated and the collector voltage (*V*_C_) increases. As the *V*_C_ reaches the *V*_LU_, the accumulated charges flow through the base and the *V*_C_ returns to the *V*_LD_. This voltage oscillation is repeated as the closed-loop feedback during the current is applied.

**Figure S6. Oscillation frequency of the biristor according to the biristor capacitor.**

Figure S6 illustrates the measured and simulated oscillation frequency of the InGaAs biristor according to the biristor capacitance. This tendency can be represented by the following equation^[6]^,

| $f_{osc}=\frac{I_{\mathrm{in}}}{C_{\mathrm{par}}(V_{\mathrm{LU}}-V_{\mathrm{LD}})}$ | (1) |
| --- | --- |

where *C*_par_ denotes the parasitic capacitance, *V*_LU_ represents the latch-up voltage and *V*_LD_ is the latch-down voltage. As the biristor scaled down, its capacitance also decreased, leading to a proportional increase in the oscillation frequency.^[7]^ In this experiment, the effects of unintended parasitic capacitance caused by the measurement setup, including the oscilloscope, parameter analyzer, and cables, limit the reduction in capacitance even when scaling down the size of the biristor. However, in practical applications, the developed device would be implemented as part of a CMOS-integrated Ising chip, where all components, including the SHIL generation and phase-readout circuits, would be integrated into a single chip. This integration eliminates the need for bulky external equipment, significantly reducing parasitic capacitance. As a result, in practical use, scaling down the size of the biristor is expected to lead to a decrease in the capacitance of the device, which in turn increases the oscillation frequency. Additionally, as the active area of the biristor decreases, the latch process occurs more rapidly, reducing the latch window and further increasing the frequency. Therefore, based on SPICE modeling and simulation, when the biristor capacitance is scaled down to the pF range, GHz-level operation becomes achievable in the InGaAs biristor.

**Figure S7. Settling cycles to the locking phase according to the coupling resistor.**

**Figure S8. The experimental measurement setup for the fabricated M3D oscillatory Ising machine.**

The measurement setup includes a function generator, oscilloscope, Keithley 4200 semiconductor parameter analyzer, oscilloscope, and a custom printed circuit board (PCB) equipped with the fabricated M3D Ising machine array. The M3D Ising machine array is mounted at the center of the PCB, with the metal pads of the biristors wire-bonded and connected to the PCB pads.

**Figure S9. (a) The measurement schematic of the SHIL in the biristor. (b) Output waveforms of the SHIL and biristor when the biristor is locked to the SHIL, showing two random phases (blue or red)**

The SHIL helps the coupled oscillator network to settle into the phase of the oscillator into one of the two steady phases. The schematic for the measurement of SHIL in the biristor is shown in **Figure. S9(a)**. As we adjusted the *I*_in_ to 250 μA to attain a frequency of the biristor (*f*_osc_) of approximately 260 kHz and introduced a sinusoidal wave with an injection capacitance (*C*_inj_) of 20 pF, setting 2*f*_osc_ ≈ *f*_inj_ results in the oscillator's phase converging to 0° or 180°. Notably, during SHIL, the single biristor’s waveform appears in two different phases (red and blue in **Figure S9(b)** with equal probability.

**Figure S10. (a) Programmed weight schematic of the six-routing switches and four-biristors King’s graph block. (b) The measured output waveforms of the biristors (*I*_in_ = 250 μA)**

**Figure S11. Cross-sectional schematic of a (a) 2D planar configuration and (b) M3D integrated configuration for a biristor coupled with a FeFET-MIM capacitor. (c) The simulation results compare the frequency of the configured biristor array for the number of biristors.**

In the 2D planar structure, the simulation result shows that the frequency of the biristor in the array decreases due to the influence of parasitic capacitance from metal lines. This effect becomes more pronounced as the number of oscillators in the array increases, making the array more complex. In contrast, in the M3D structure, since the biristors are integrated on top of the FEOL, they are less affected by wire parasitics, which helps mitigate frequency degradation.

**References**

[1] S. Dutta, A. Khanna, A. Assoa, H. Paik, D. G. Schlom, Z. Toroczkai, A. Raychowdhury, S. Datta, *Nature Electronics* **2021**, *4* (7), 502.

[2] S. Boixo, T. F. Rønnow, S. V. Isakov, Z. Wang, D. Wecker, D. A. Lidar, J. M. Martinis, M. Troyer, *Nature physics* **2014**, *10* (3), 218.

[3] R. Hamerly, T. Inagaki, P. L. McMahon, D. Venturelli, A. Marandi, T. Onodera, E. Ng, C. Langrock, K. Inaba, T. Honjo, *Science advances* **2019**, *5* (5), eaau0823.

[4] J. Bae, J. Koo, C. Shim, B. Kim, in *2024 IEEE International Solid-State Circuits Conference (ISSCC)* IEEE, **2024**, 284-286.

[5] F. Cai, S. Kumar, T. Van Vaerenbergh, X. Sheng, R. Liu, C. Li, Z. Liu, M. Foltin, S. Yu, Q. Xia, *Nature Electronics* **2020**, *3* (7), 409.

[6] J.-K. Han, M. Seo, W.-K. Kim, M.-S. Kim, S.-Y. Kim, M.-S. Kim, G.-J. Yun, G.-B. Lee, J.-M. Yu, Y.-K. Choi, *IEEE Electron Device Letters* **2019**, *41* (2), 208.

[7] J.-W. Han, Y.-K. Choi, *IEEE Electron Device Letters* **2010**, *31* (8), 797.
